# Application of Artificial Intelligence for Screening COVID-19 Patients Using Digital Images: Meta-analysis

**DOI:** 10.2196/21394

**Published:** 2021-04-29

**Authors:** Tahmina Nasrin Poly, Md Mohaimenul Islam, Yu-Chuan Jack Li, Belal Alsinglawi, Min-Huei Hsu, Wen Shan Jian, Hsuan-Chia Yang

**Affiliations:** 1 Graduate Institute of Biomedical Informatics College of Medical Science and Technology Taipei Medical University Taipei Taiwan; 2 International Center for Health Information Technology Taipei Medical University Taipei Taiwan; 3 Research Center of Big Data and Meta-Analysis Wan Fang Hospital Taipei Medical University Taipei Taiwan; 4 Department of Dermatology Wan Fang Hospital Taipei Medical University Taipei Taiwan; 5 TMU Research Center of Cancer Translational Medicine Taipei Medical University Taipei Taiwan; 6 School of Computer, Data, and Mathematical Science Western Sydney University Sydney Australia; 7 Graduate Institute of Data Science Taipei Medical University Taipei Taiwan; 8 School of Health Care Administration Taipei Medical University Taipei Taiwan

**Keywords:** COVID-19, SARS-CoV-2, pneumonia, artificial intelligence, deep learning

## Abstract

**Background:**

The COVID-19 outbreak has spread rapidly and hospitals are overwhelmed with COVID-19 patients. While analysis of nasal and throat swabs from patients is the main way to detect COVID-19, analyzing chest images could offer an alternative method to hospitals, where health care personnel and testing kits are scarce. Deep learning (DL), in particular, has shown impressive levels of performance when analyzing medical images, including those related to COVID-19 pneumonia.

**Objective:**

The goal of this study was to perform a systematic review with a meta-analysis of relevant studies to quantify the performance of DL algorithms in the automatic stratification of COVID-19 patients using chest images.

**Methods:**

A search strategy for use in PubMed, Scopus, Google Scholar, and Web of Science was developed, where we searched for articles published between January 1 and April 25, 2020. We used the key terms “COVID-19,” or “coronavirus,” or “SARS-CoV-2,” or “novel corona,” or “2019-ncov,” and “deep learning,” or “artificial intelligence,” or “automatic detection.” Two authors independently extracted data on study characteristics, methods, risk of bias, and outcomes. Any disagreement between them was resolved by consensus.

**Results:**

A total of 16 studies were included in the meta-analysis, which included 5896 chest images from COVID-19 patients. The pooled sensitivity and specificity of the DL models in detecting COVID-19 were 0.95 (95% CI 0.94-0.95) and 0.96 (95% CI 0.96-0.97), respectively, with an area under the receiver operating characteristic curve of 0.98. The positive likelihood, negative likelihood, and diagnostic odds ratio were 19.02 (95% CI 12.83-28.19), 0.06 (95% CI 0.04-0.10), and 368.07 (95% CI 162.30-834.75), respectively. The pooled sensitivity and specificity for distinguishing other types of pneumonia from COVID-19 were 0.93 (95% CI 0.92-0.94) and 0.95 (95% CI 0.94-0.95), respectively. The performance of radiologists in detecting COVID-19 was lower than that of the DL models; however, the performance of junior radiologists was improved when they used DL-based prediction tools.

**Conclusions:**

Our study findings show that DL models have immense potential in accurately stratifying COVID-19 patients and in correctly differentiating them from patients with other types of pneumonia and normal patients. Implementation of DL-based tools can assist radiologists in correctly and quickly detecting COVID-19 and, consequently, in combating the COVID-19 pandemic.

## Introduction

COVID-19 is a serious global infectious disease and is spreading at an unprecedented level worldwide [1,2]. The World Health Organization declared this infectious disease a public health emergency of international concern and then declared it a pandemic. SARS-CoV-2 is even more contagious than SARS-CoV or Middle East respiratory syndrome coronavirus and is sometimes undetected due to people having asymptomatic or mild symptoms [3,4]. Earlier detection paired with aggressive public health steps, such as social distancing and isolation of suspected or sick patients, can help tackle the crisis [5]. Presently, reverse transcription–polymerase chain reaction (RT-PCR), gene sequencing, and analysis of blood specimens are considered the gold standard methods for detecting COVID-19; however, the performance of these methods (∼73% sensitivity for nasal swabs and ∼61% for throat swabs) is not satisfactory [6,7]. Since hospitals are overwhelmed by COVID-19 patients, those with severe acute respiratory illness are given priority over others with mild symptoms. Therefore, a large number of undiagnosed patients may lead to a serious risk of cross-infection.

Chest radiography imaging (eg, x-ray and computed tomography [CT] scan) is often used as an effective tool for the quick diagnosis of pneumonia [8,9]. The CT scan images of COVID-19 patients show multilobar involvement and peripheral airspace, mostly ground-glass opacities [10,11]. Moreover, asymmetric patchy or diffuse airspace opacities have also been reported in patients with SARS-CoV-2 infection [12]. These changes in CT scan images can be easily interpreted by a trained or experienced radiologist. Automatic classification of COVID-19 patients, however, has huge benefits, such as increasing efficiency, wide coverage, reducing barriers to access, and improving patient outcomes. Several studies demonstrated the application of deep learning (DL) techniques to identify and detect novel COVID-19 using radiography images [13,14].

Herein, we report the results of a comprehensive systematic review of DL algorithm studies that investigated the performance of DL algorithms for COVID-19 classification from chest radiography imaging. Our main objective was to quantify the performance of DL methods for COVID-19 classification, which might encourage health care policy makers to implement DL-based automated tools in the real-world clinical setting. DL-based automated tools can help reduce radiologists’ workload, as DL can help maintain diagnostic radiology support in real time and with increased sensitivity.

## Methods

### Experimental Approach

The PRISMA (Preferred Reporting Items for Systematic Reviews and Meta-Analyses) guidelines, which are based on the Cochrane Handbook for Systematic Reviews of Interventions, were used to conduct this study [15].

### Literature Search

We searched electronic databases, such as PubMed, Scopus, Google Scholar, and Web of Science, for articles published between January 1 and April 25, 2020. We developed a search strategy using combinations of the following Medical Subject Headings: “COVID-19,” or “coronavirus,” or “SARS-CoV-2,” or “novel corona,” or “2019-ncov,” and “deep learning,” or “artificial intelligence,” or “automatic detection.” Reference lists of the retrieved articles and relevant reviews were also checked for additional eligible articles.

### Eligibility Criteria

During the first screening, two authors (MMI and TNP) assessed the title and abstract of each article and excluded irrelevant articles. To include eligible articles, those two authors examined the full text of the articles and evaluated whether they fulfilled the inclusion criteria of this study. Disagreement during this selection process was resolved by consensus or, if necessary, the main investigator (YCL) was consulted. We included articles if they met the following criteria: (1) were published in English, (2) were published in a peer-reviewed journal, (3) assessed performance of a DL model to detect COVID-19, and (4) provided a clear description of the methodology and the total number of images. We excluded studies if they were published in preprint repositories or if they were published in the form of a review or a letter to the editor.

### Data Extraction and Synthesis

Two authors (MMI and TNP) independently screened all titles and abstracts of retrieved articles. The most relevant studies were selected based on the predefined selection criteria. Any disagreement during the screening process was resolved by discussion with the other authors; unsettled issues were settled by discussion with the study supervisor (YCL). The two authors who conducted the first screening cross-checked studies for duplication by comparing author names, publication dates, and journal names. They excluded all duplicate studies. Afterward, they collected data from the selected studies, such as author name, publication year, location, model description, total number of images, total number of COVID-19 cases and images, imaging modality, total number of patients, sensitivity, specificity, accuracy, area under the receiver operating characteristic curve (AUROC), and database.

### Risk of Bias Assessment

The Quality Assessment of Diagnostic Accuracy Studies-2 (QUADAS-2) tool was used to assess the quality of the selected studies [16]. The QUADAS-2 scale comprises four domains: patient selection, index test, reference standard, and flow and timing. The first three domains are used to evaluate the risk of bias in terms of concerns regarding applicability. The overall risk of bias was categorized into three groups: low, high, and unclear risk of bias.

### Statistical Analysis

Meta-DiSc, version 1.4, was used to calculate the evaluation metrics of the DL model. The software was also used to (1) perform statistical pooling from each study and (2) assess the homogeneity with a variety of statistics, including chi-square and I^2^. The sensitivity and specificity with 95% CIs in distinguishing between COVID-19 patients, patients with other types of pneumonia, and normal patients were calculated. The pooled receiver operating characteristic (ROC) curve was plotted and the area under the curve (AUC) was calculated with 95% CIs based on the DerSimonian-Laird random effects model method. The diagnostic odds ratio (DOR) was calculated by the Moses constant of the linear model. Diagnostic tests where the DOR is constant, regardless of the diagnostic threshold, have symmetrical curves around the sensitivity-specificity line. In these situations, it is possible to combine DORs using the DerSimonian-Laird method to estimate the overall DOR and, hence, to determine the best-fitting ROC curve [17]. The mathematical equation is given below:


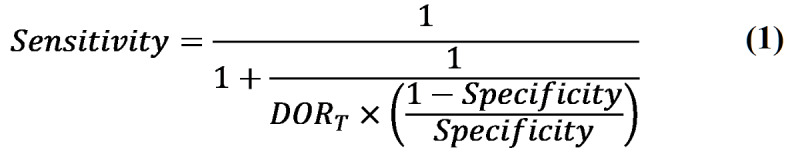


When the DOR changes with the diagnostic threshold, the ROC curve is asymmetrical. To fit the DOR variation based on a different threshold, the Moses-Shapiro-Littenberg method was used. It consists of observing the relationship by fitting the straight line:

*D* = *a* + *bS*        **(2)**

where *D* is the log of DOR and *S* is a measure of threshold given by the following:


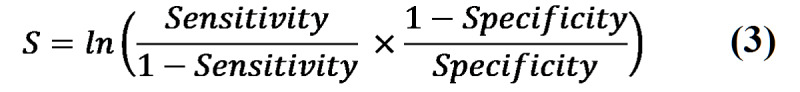


Estimates of parameters *a* and *b* and their standard errors and covariance were obtained by the ordinary or weighted least squares method using the NAG Library for C (The Numerical Algorithms Group).

The ROC curve is the AUC that summarized the diagnostic performance as a single number: an AUC close to 1 is considered a perfect curve and an AUC close to 0.5 is considered poor [18]. The AUC is computed by numeric integration of the curve equation by the trapezoidal method [19]. The Q* index is defined by the point where sensitivity and specificity are equal, which is the point closest to the ideal top-left corner of the ROC curve space. It was calculated by the following:


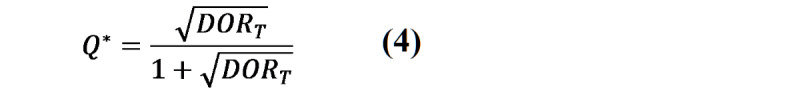


Moreover, the standard error of the AUROC was calculated by following equation:


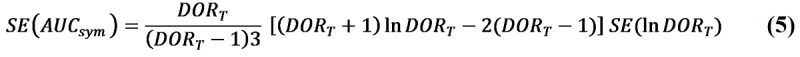


The standard error of Q* was calculated by following equation:


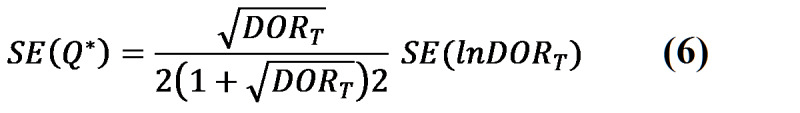


## Results

### Selection Criteria

**Figure 1:**
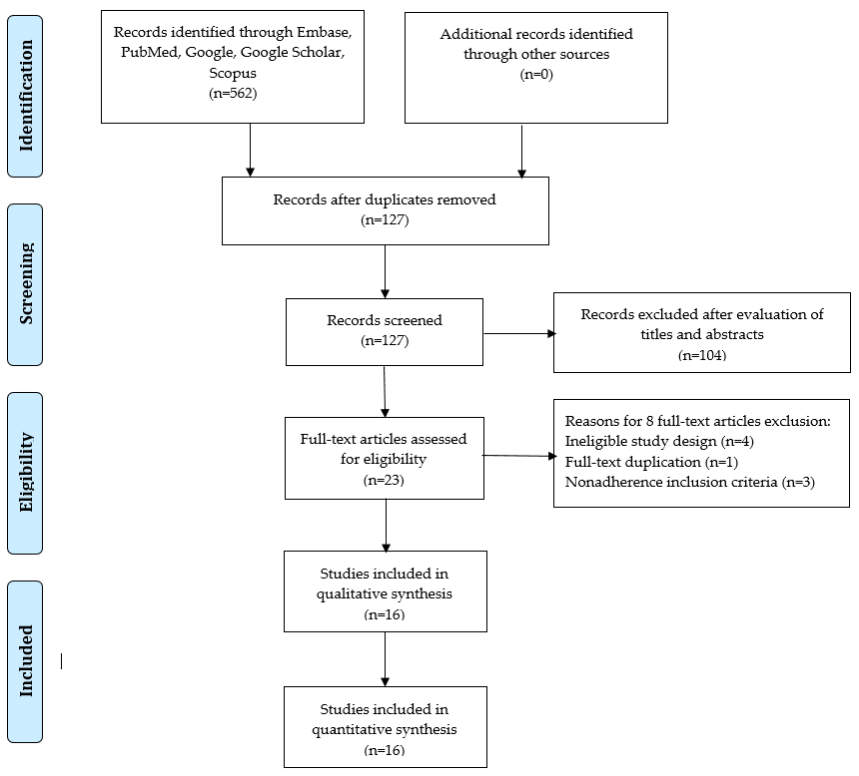
PRISMA (Preferred Reporting Items for Systematic Reviews and Meta-Analyses) flow diagram for study selection.

Figure 1 shows the process of identifying relevant DL studies. A total of 562 studies were retrieved by searching electronic databases and by reviewing their reference lists. We excluded 435 duplicate studies and an additional 104 studies that did not fulfill the selection criteria. We reviewed 23 full-text studies and further excluded 7 studies because of the reasons shown in Figure 1. Finally, we included 16 studies in the meta-analysis [13,14,20-33].

### Characteristics of Included Studies

Among the 16 DL-based COVID-19 detection studies, we identified 5896 digital images for COVID-19 patients and 645,825 images for non-COVID-19 patients, including those with other types of viral pneumonia and normal patients. Included studies used DL algorithms, such as convolutional neural networks, MobileNetV2, and COVNet, for stratifying COVID-19 patients with higher accuracy. The range of accuracy for detecting COVID-19 correctly was 76.00 to 99.51. A total of 8 studies used CT images and 8 studies used x-ray images. The characteristics of the included studies in the meta-analysis are shown in Table 1 [13,14,20-33].

**Table 1:** Characteristics of the studies included in the meta-analysis.

Author	Modality	Method	Images, n	COVID-19 images, n	Sensitivity	Specificity	Accuracy
Apostolopoulos and Mpesiana [14]	X-ray	MobileNetV2	1428	224	98.66	96.46	99.18
Butt et al [13]	Computed tomography (CT)	Convolutional neural network (CNN)	618	219	98.20	92.20	—^a^
Apostolopoulos et al [21]	X-ray	MobileNetV2	3905	463	97.36	99.42	96.78
Li et al [25]	CT	COVNet	4356	127	90.00	95.00	—
Ucar and Korkmaz [29]	X-ray	CNN	4608^b^	1536^b^	—	99.13	98.30
Ozturk et al [26]	X-ray	CNN and DarkNet	1186	108	95.13	95.30	98.08
Bai et al [24]	CT	EfficientNet	1186	521	95.00	96.00	96.00
Zhang at al [33]	CT	DeepLabv3	617,775	—	94.93	91.13	92.49
El Asnaoui and Chawki [20]	X-ray	Inception ResNet V2	6087	231	92.11	96.06	—
Ardakani et al [22]	CT	ResNet-101	1020	510	100	99.02	99.51
Pathak et al [27]	CT	CNN	852	413	91.45	94.77	93.01
Wu et al [32]	CT	ResNet50	495	368	81.10	61.50	76.00
Toğaçar et al [28]	X-ray	SqueezeNet	458	295	100	100	100
Waheed at al [30]	X-ray	ACGAN^c^	1124	403	90.00	97.00	95.00
Khan et al [23]	X-ray	Xception	1251	284	99.30	98.60	99.00
Wang et al [31]	CT	DenseNet	5372	102	80.39	76.66	78.32
Wang et al [31]	CT	DenseNet	5372	92	79.35	81.16	80.12

^a^Not reported.

^b^Augmented images.

^c^ACGAN: auxiliary classifier generative adversarial network.

### Model Performance

**Figure 2:**
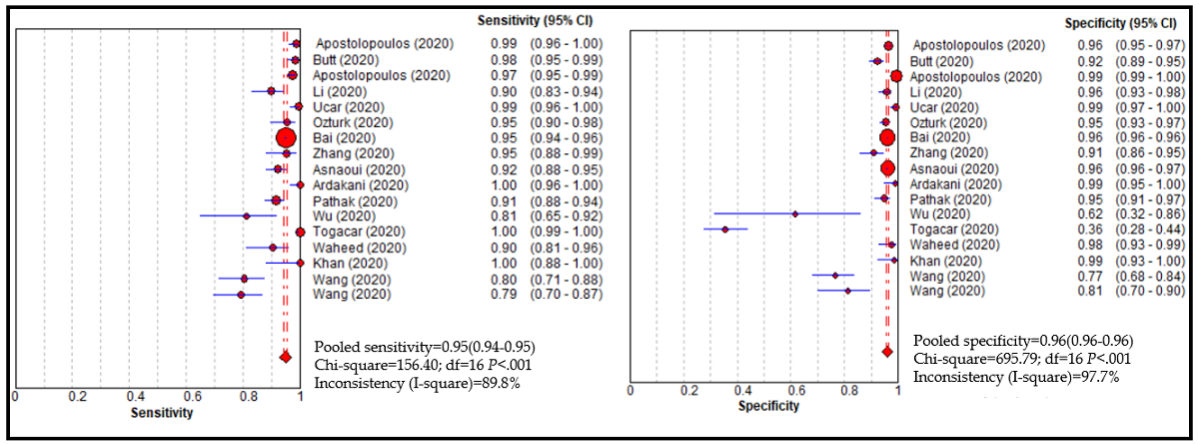
Performance of the deep learning model for detecting COVID-19.

**Figure 3:**
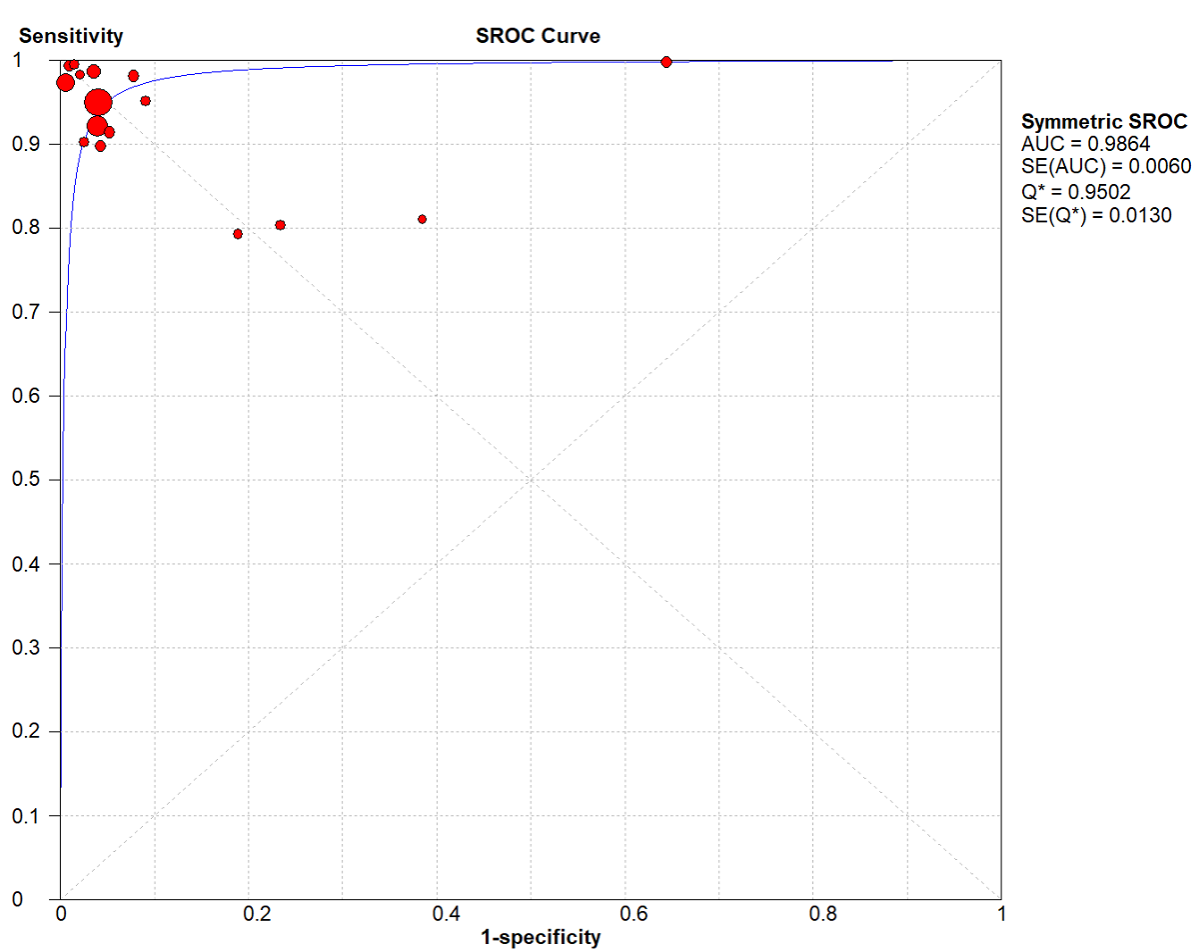
Summary receiver operating characteristic (SROC) curve of the deep learning method. AUC: area under the curve; Q*: this index is defined by the point where sensitivity and specificity are equal.

Based on the 16 studies, the performance of the DL algorithms for detecting COVID-19 was determined and is summarized in Table 2 [22,24,33]. The pooled sensitivity and specificity of the DL methods for detecting COVID-19 was 0.95 (95% CI 0.94-0.95) and 0.96 (95% CI 0.96-0.97), respectively, with a summary ROC (SROC) of 0.98 (Figure 2). The pooled sensitivity and specificity are shown in Figure 3.

DL methods were able to correctly distinguish other types of pneumonia from COVID-19 with an SROC of 0.98 (sensitivity: 0.93, 95% CI 0.92-0.94; specificity: 0.95, 95% CI 0.94-0.95). The positive likelihood, negative likelihood, and DOR were 22.45 (95% CI 12.86-39.19), 0.06 (95% CI 0.03-0.13), and 461.81 (95% CI 134.96-1580.24), respectively. Moreover, the DL model showed good performance for correctly stratifying normal patients, with an SROC of 0.99 (sensitivity: 0.95, 95% CI 0.94-0.96; specificity: 0.98, 95% CI 0.97-0.98). The positive likelihood, negative likelihood, and DOR were 47.47 (95% CI 20.70-108.86), 0.04 (95% CI 0.02-0.08), and 1524.81 (95% CI 625.29-3718.34), respectively.

**Table 2:** Performance comparison between deep learning models and radiologists.

Class and method					Data sets, n			Sensitivity (95% CI)			Specificity (95% CI)			Positive likelihood ratio (95% CI)			Negative likelihood ratio (95% CI)			AUROC^a^			Accuracy			
**COVID-19**																										
	Deep learning model			17			0.95 (0.94-0.95)			0.96 (0.96-0.97)			19.02 (12.83-28.19)			0.06 (0.04-0.10)			0.98			—^b^				
	**Radiologists** **(Bai et al [24])**																									
		Total	6			0.79 (0.64-0.89)			0.88 (0.78-0.94)			—			—			—			0.85					
		Junior^c^	3			0.80 (0.72-0.87)			0.88 (0.83-0.92)			—			—			—			—					
		Senior^d^	3			0.78 (0.70-0.85)			0.87 (0.82-0.91)			—			—			—			—					
		Junior + AI^e^	—			0.88 (0.81-0.93)			0.93 (0.89-0.96)			—			—			—			—					
		Senior + AI	—			0.88 (0.81-0.93)			0.89 (0.84-0.93)			—			—			—			—					
	**Radiologists (Zhang et al [33])**																									
		Total	8			0.75 (0.65-0.84)			0.90 (0.86-0.94)			—			—			—			—					
		Junior	4			0.65 (0.48-0.79)			0.89 (0.81-0.94)			—			—			—			0.82					
		Senior	4			0.85 (0.70-0.94)			0.91 (0.85-0.96)			—			—			—			0.90					
		Junior + AI	—			0.80 (0.64-0.90)			0.94 (0.88-0.97)			—			—			—			0.90					
	Radiologist (Ardakani et al [22]; senior)			1			0.89 (0.81-0.94)			0.83 (0.74-0.89)			—			—			—			—				
Other types of pneumonia: deep learning model					7			0.93 (0.92-0.94)			0.95 (0.94-0.95)			22.45 (12.86-39.19)			0.06 (0.03-0.13)			0.98			—			
Normal: deep learning model					6			0.95 (0.94-0.96)			0.98 (0.97-0.98)			47.47 (20.70-108.86)			0.04 (0.02-0.08)			0.99			—			

^a^AUROC: area under the receiver operating characteristic curve.

^b^Not reported.

^c^Junior radiologists have 5 to 15 years of experience.

^d^Senior radiologists have 15 to 25 years of experience.

^e^AI: artificial intelligence.

### Performance of Radiologists

#### Overview

A total of 3 studies compared the performance of DL models with radiologists [22,24,33]. Zhang et al [33] included 8 radiologists with 5 to 25 years of experience; they were categorized into two groups: junior radiologists had 5 to 15 years of experience and senior radiologists had 15 to 25 years of experience. Bai et al [24] compared DL model performance with 6 radiologists; 3 of them had 10 years of experience (ie, junior) and 3 had 20 years of experience (ie, senior). Finally, Ardakani et al [22] compared the performance of DL models with 1 senior radiologist, who had 15 years of experience. The performance of 15 radiologists in detecting COVID-19 was evaluated; the pooled sensitivity and specificity for detecting COVID-19 ranged from 0.75 to 0.89 and from 0.83 to 0.90, respectively. With the assistance of DL-based artificial intelligence (AI) tools, the performance of the junior radiologists improved: sensitivity improved by 0.08 to 0.15 and specificity improved by 0.05.

#### Sensitivity Analysis

A total of 8 studies evaluated the performance of DL algorithms for detecting COVID-19 using x-ray photographs. The pooled sensitivity and specificity of DL algorithms for detecting COVID-19 were 0.96 (95% CI 0.95-0.97) and 0.97 (95% CI 0.97-0.98), respectively, with an SROC of 0.99. Moreover, 8 studies assessed the performance of DL algorithms for classifying COVID-19 using CT images. The pooled sensitivity and specificity were 0.94 (95% CI 0.94-0.95) and 0.95 (95% CI 0.95-0.96), respectively, with an SROC of 0.96 (see Figures S1-S12 in Multimedia Appendix 1).

#### Risk of Bias and Applicability

In this meta-analysis, we also assessed heterogeneous findings that originated from included studies based on the QUADAS-2 tool (see Table 3 [13,14,20-33]). The risk of bias for patient selection was unclear for 16 studies. All studies had an unclear risk of bias for flow and timing and for the index test. Moreover, all studies had a high risk of bias for the reference standard. In the case of applicability, all studies had a low risk of bias for patient selection. However, the risk of index test and the applicability concern for the reference standard were uncertain.

**Table 3:** Quality Assessment of Diagnostic Accuracy Studies-2 for included studies.

Study	Risk of bias (high, low, or unclear)					Applicability concerns		
	Patient selection	Index test	Reference standard	Flow and timing	Patient selection		Index test	Reference standard
Apostolopoulos and Mpesiana [14]	High	Unclear	High	Unclear	Low		Unclear	Unclear
Butt et al [13]	High	Unclear	High	Unclear	Low		Unclear	Unclear
Apostolopoulos et al [21]	High	Unclear	High	Unclear	Low		Unclear	Unclear
Li et al [25]	High	Unclear	High	Unclear	Low		Unclear	Unclear
Ucar and Korkmaz [29]	High	Unclear	High	Unclear	Low		Unclear	Unclear
Ozturk et al [26]	High	Unclear	High	Unclear	Low		Unclear	Unclear
Bai et al [24]	High	Unclear	High	Unclear	Low		Unclear	Unclear
Zhang at al [33]	High	Unclear	High	Unclear	Low		Unclear	Unclear
El Asnaoui and Chawki [20]	High	Unclear	High	Unclear	Low		Unclear	Unclear
Ardakani et al [22]	High	Unclear	High	Unclear	Low		Unclear	Unclear
Pathak et al [27]	High	Unclear	High	Unclear	Low		Unclear	Unclear
Wu et al [32]	High	Unclear	High	Unclear	Low		Unclear	Unclear
Toğaçar et al [28]	High	Unclear	High	Unclear	Low		Unclear	Unclear
Waheed at al [30]	High	Unclear	High	Unclear	Low		Unclear	Unclear
Khan et al [23]	High	Unclear	High	Unclear	Low		Unclear	Unclear
Wang et al [31]	High	Unclear	High	Unclear	Low		Unclear	Unclear

## Discussion

### Principal Findings

In this study, we evaluated the performance of the DL model regarding detection of COVID-19 automatically using chest images to assist with proper diagnosis and prognosis. The findings of our study showed that the DL model achieved high sensitivity and specificity (95% and 96%, respectively) when detecting COVID-19. The pooled SROC value of both COVID-19 and other types of pneumonia was 98%. The performance of the DL model was comparable to that of experienced radiologists, whose clinical experience was at least 10 years, and the model could improve the performance of junior radiologists.

### Clinical Implications

The rate of COVID-19 cases has been mounting day by day; therefore, it is important to quickly and accurately diagnose patients so that we may combat this pandemic. However, screening an increased number of chest images is challenging for the radiologists, and the number of trained radiologists is not sufficient, especially in underdeveloped and developing countries [34]. The recent success of DL applications in imaging analysis of CT scans, as well as x-ray imaging in automatic segmentation and classification in the radiology domain, has encouraged health care providers and researchers to exploit the advancement of deep neural networks in other applications [35]. DL models have been trained to assist radiologists in achieving higher interrater reliability during their years of experience in clinical practice.

Since the start of the COVID-19 pandemic, efforts have been made by AI researchers and AI modelers to help radiologists in the rapid diagnosis of COVID-19 in order to combat the COVID-19 pandemic [33,36]. Developing an accurate, automated AI COVID-19 detection tool is deemed as essential in reducing unnecessary waiting times, shortening screening and examination times, and improving performance. Moreover, such a tool could help to reduce radiologists’ workloads and allow them to respond to emergency situations rapidly and in a cost-effective manner [25]. RT-PCR is considered the gold standard detection method; however, findings of our study showed that chest CT could be used as a reliable and rapid approach for screening of COVID-19. Our findings also showed that the DL model was able to discriminate COVID-19 from other types of pneumonia with high a sensitivity and specificity, which is a challenging task for radiologists [32].

### Strengths and Limitations

Our study has several strengths. First, this is the first meta-analysis that evaluated the performance of a DL model to classify COVID-19 patients. Second, we considered only peer-reviewed articles to be included in our study because articles that are not peer reviewed might contain bias. Third, we compared the performance of the DL model with that of senior and junior radiologists, which would be helpful for policy makers in considering an automated classification system in real-world clinical settings in order to speed up routine examination.

However, our study also has some limitations that need to be addressed. First, only 16 studies were used to evaluate the performance of the model; inclusion of more studies may have provided more specific findings. Second, some studies included similar data sets, which may have created some bias, but the researchers in those studies had optimized algorithms to improve performance. Third, two different kinds of digital photographs (ie, CT scan and x-ray) were used to develop and evaluate the performance of the DL model in classifying COVID-19; however, the performance of the DL model was almost the same in both cases. Finally, none of the studies included external validation; therefore, model performance could vary if those models were implemented in other clinical settings.

### Future Perspective

The primary objectives of prediction models are the quick screening of COVID-19 patients and to help physicians make appropriate decisions. Misdiagnosis could have a destructive effect on society, as COVID-19 could spread from infected people to healthy people. Therefore, it is important to select a target population among which this automated tool could serve a clinical need; it is also important to select a representative data set on which the model could be trained, developed, and validated internally and externally. All the studies included in this meta-analysis had a high risk of bias for patient selection and reference standards. Moreover, generalizability was lacking in the newly developed classification models. Models without proper evidence and with a lack of external validation are not appropriate for clinical practice because they might cause more harm than good. Since the number of cases is mounting each day and COVID-19 is spreading to all continents, it is therefore important to develop a model to assist in the quick and efficient screening of patients during the COVID-19 pandemic. This could encourage clinicians and policy makers to prematurely implement prediction models without sufficient documentation and validation. All studies showed promising discrimination in their training, testing, and validation cohorts, but future studies should focus on external validation and comparing their findings to other data sets. Interpretability of DL systems is more important to a health care professional than to an AI expert. Proper interpretation and explanation of algorithms will more likely be acceptable to physicians. More clinical research is needed to determine the tangible benefits for patients in terms of the high performance of the model. High sensitivity and specificity do not necessarily represent clinical efficacy, and the higher value of the AUROC is not always the best metric to exhibit clinical applicability. All papers should follow standard guidelines and they should present positive and negative predictive values in order to be able to make a fair comparison. Although all of the included studies used a significant amount of data to compare model performance to that of the radiologists, they used only retrospective data to train the models, which might result in worse performance in real-world clinical settings, as data complexity is different. Therefore, prospective evaluation is needed in future studies before considering implementation in clinical settings. AI models always consist of potential flaws, including the inapplicability of new data, reliability, and bias. Generalization of the model is important for presenting the real performance because the rate of sensitivity and specificity varied across the studies (0.79 to 1.00 and 0.62 to 1.00, respectively). A higher number of false negatives will make the situation worse and will waste health care resources.

### Conclusions

Our study showed that the DL model had immense potential to distinguish COVID-19 patients, with high sensitivity and specificity, from patients with other types of pneumonia and normal patients. DL-based tools could assist radiologists in the fast screening of COVID-19 and in classifying potential high-risk patients, which could have clinical significance for the early management of patients and could optimize medical resources. A higher number of false negatives could have a devastating effect on society; therefore, it is crucial to test the performance of models with other, unknown data sets. Retrospective evaluation and reliable interpretation are warranted to consider the application of AI models in real-world clinical settings.

## Appendix

Multimedia Appendix 1 Supplementary figures.

## Abbreviations

AI: artificial intelligence
AUC: area under the curve
AUROC: area under the receiver operating characteristic curve
CT: computed tomography
DL: deep learning
DOR: diagnostic odds ratio
MOE: Ministry of Education
MOST: Ministry of Science and Technology
PRISMA: Preferred Reporting Items for Systematic Reviews and Meta-Analyses
QUADAS-2: Quality Assessment of Diagnostic Accuracy Studies-2
ROC: receiver operating characteristic
RT-PCR: reverse transcription–polymerase chain reaction
SROC: summary receiver operating characteristic
