# Supplementary material for: Application of Artificial Intelligence for Screening COVID-19 Patients Using Digital Images: Meta-analysis
Source: JMIR Med Inform. 2021 Apr 29;9(4):e21394. doi: 10.2196/21394 (PMC8086786; doi:10.2196/21394)
Supplement: Multimedia Appendix 1 [file medinform_v9i4e21394_app1.docx]

Application of Deep Learning Model for Accurate Diagnosis of COVID-19: A Meta-Analysis

Tahmina Nasrin **Poly**^1, 2,5^; Md. Mohaimenul **Islam**^1,2,5^; Belal **Alsinglawi**^6^; Min-Huei **Hsu**^7^; Wen-Shan **Jian**^8^; Hsuan-Chia **Yang**^1,2,5,ϕ,*^;Yu-Chuan **(Jack) Li**^1, 2, 3, 4,5,ϕ^*


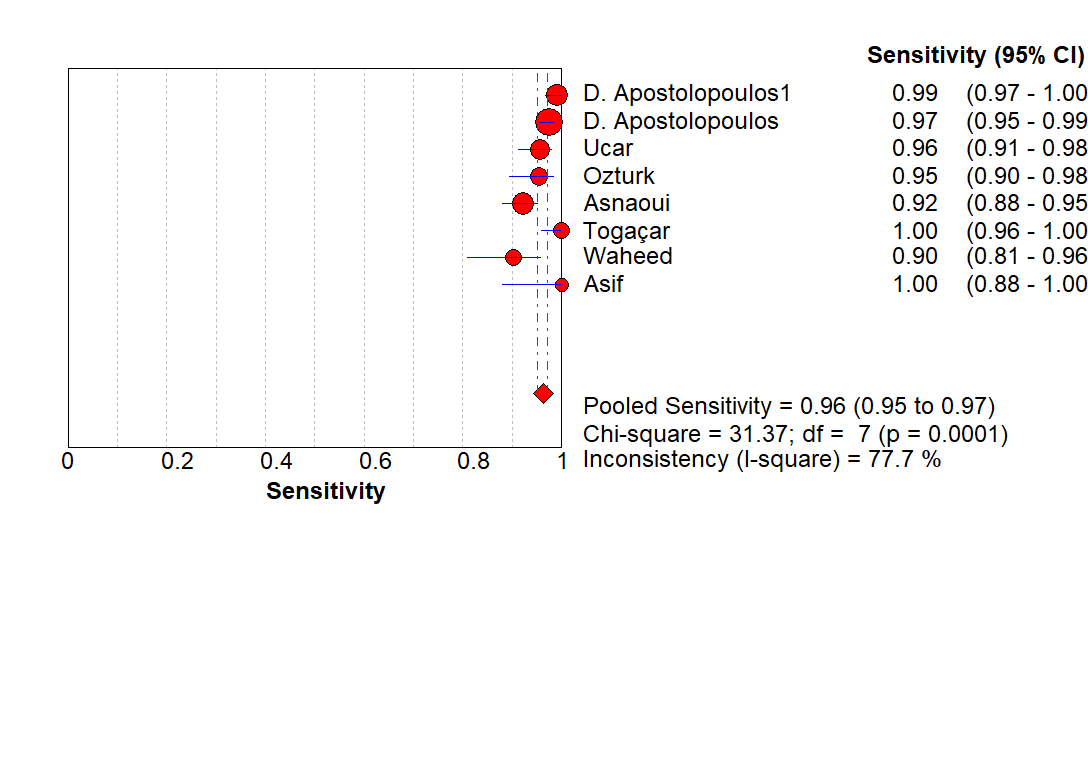


**Figure S1**: Sensitivity of DL model for COVID-19 classification using X-ray images


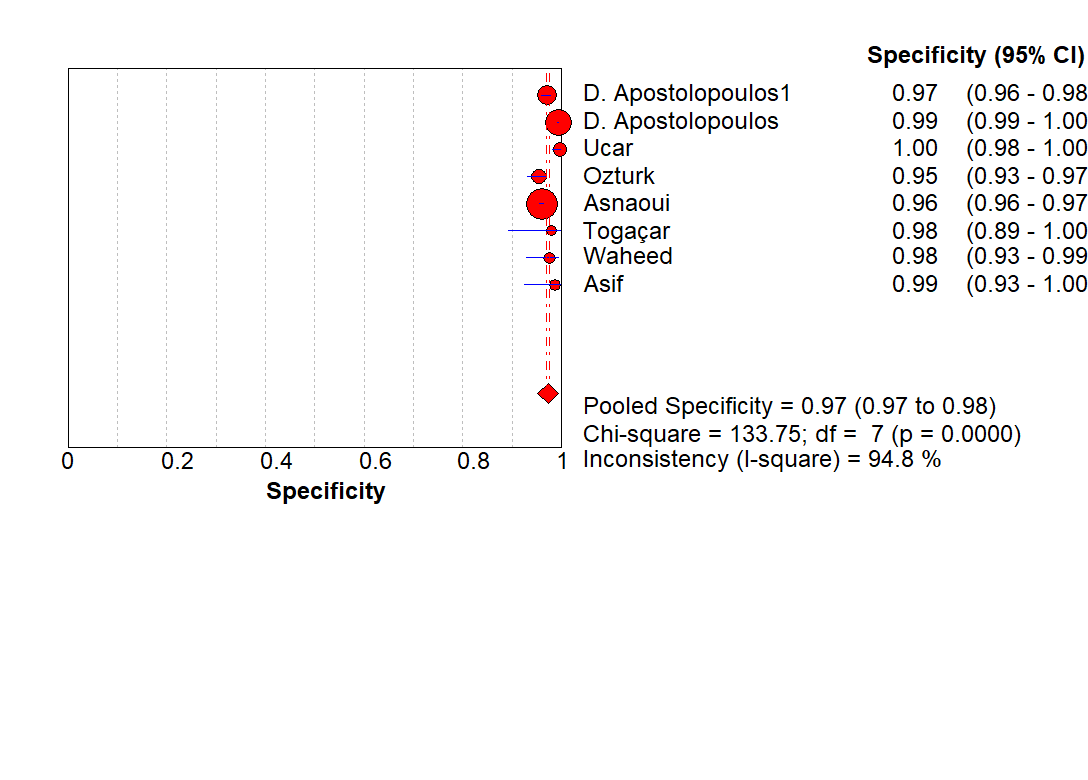


**Figure S2**: Specificity of DL model for COVID-19 classification using X-ray images


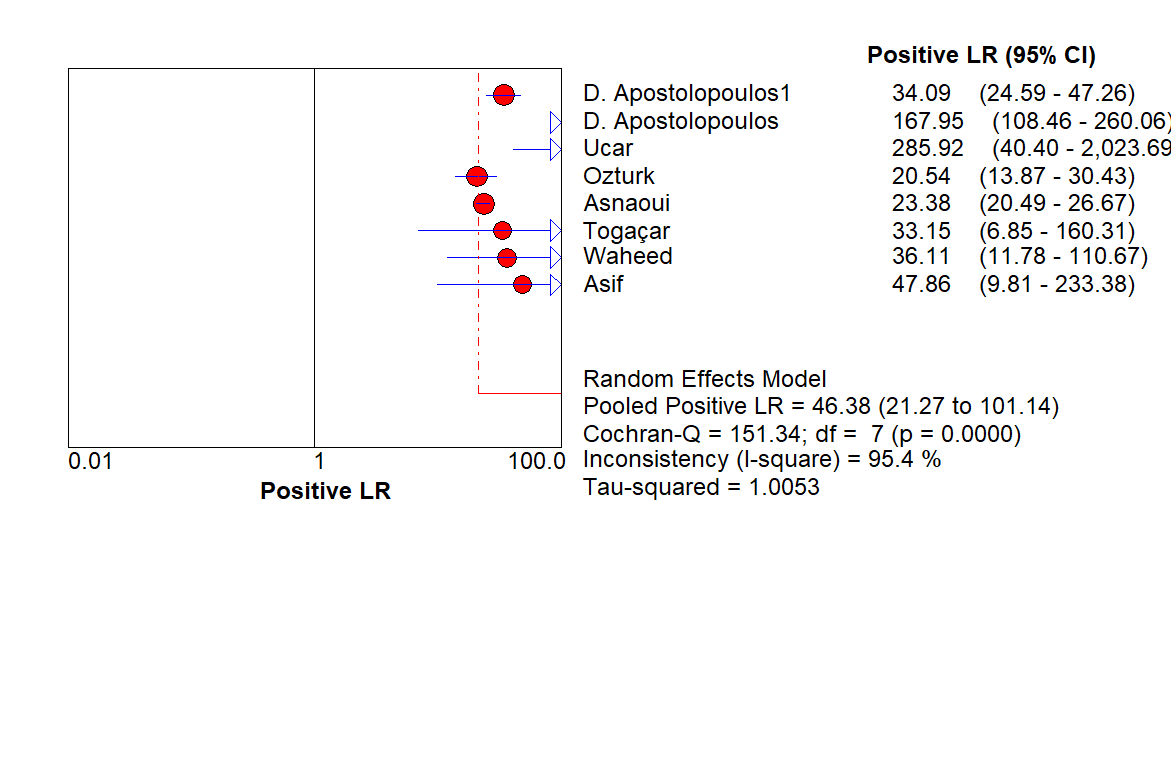


**Figure S3:** Positive LR of DL model for COVID-19 classification using X-ray images.


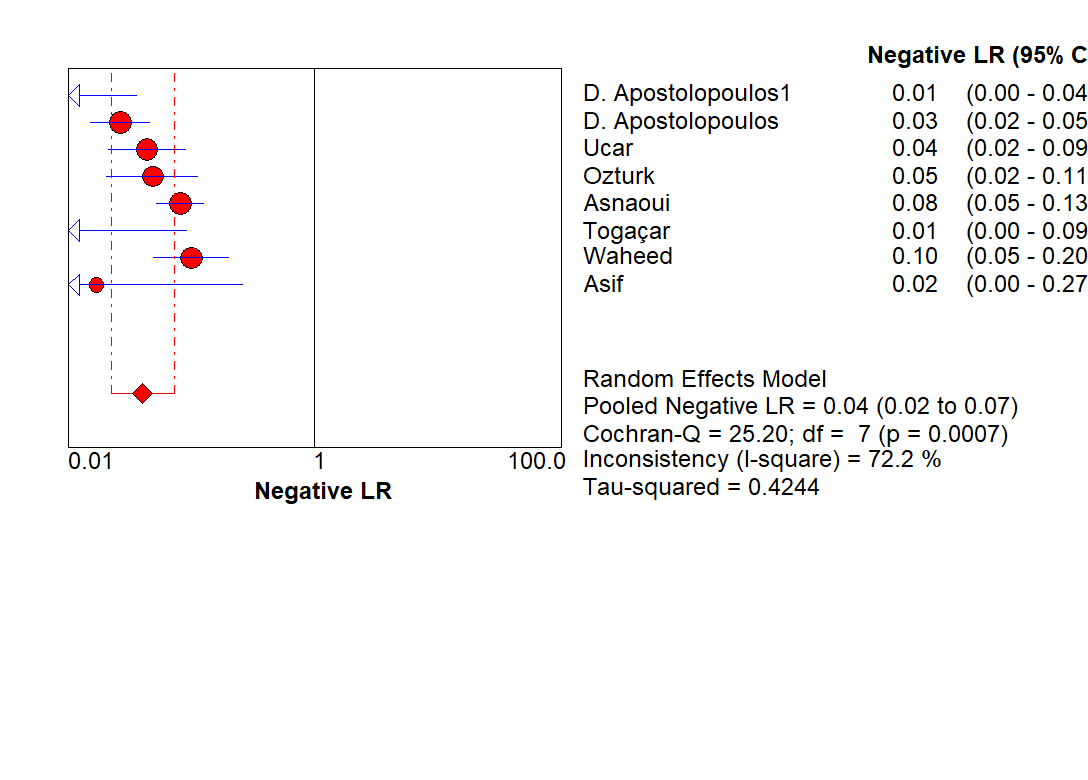


**Figure S4**: Negative LR of DL model for COVID-19 classification using X-ray images.


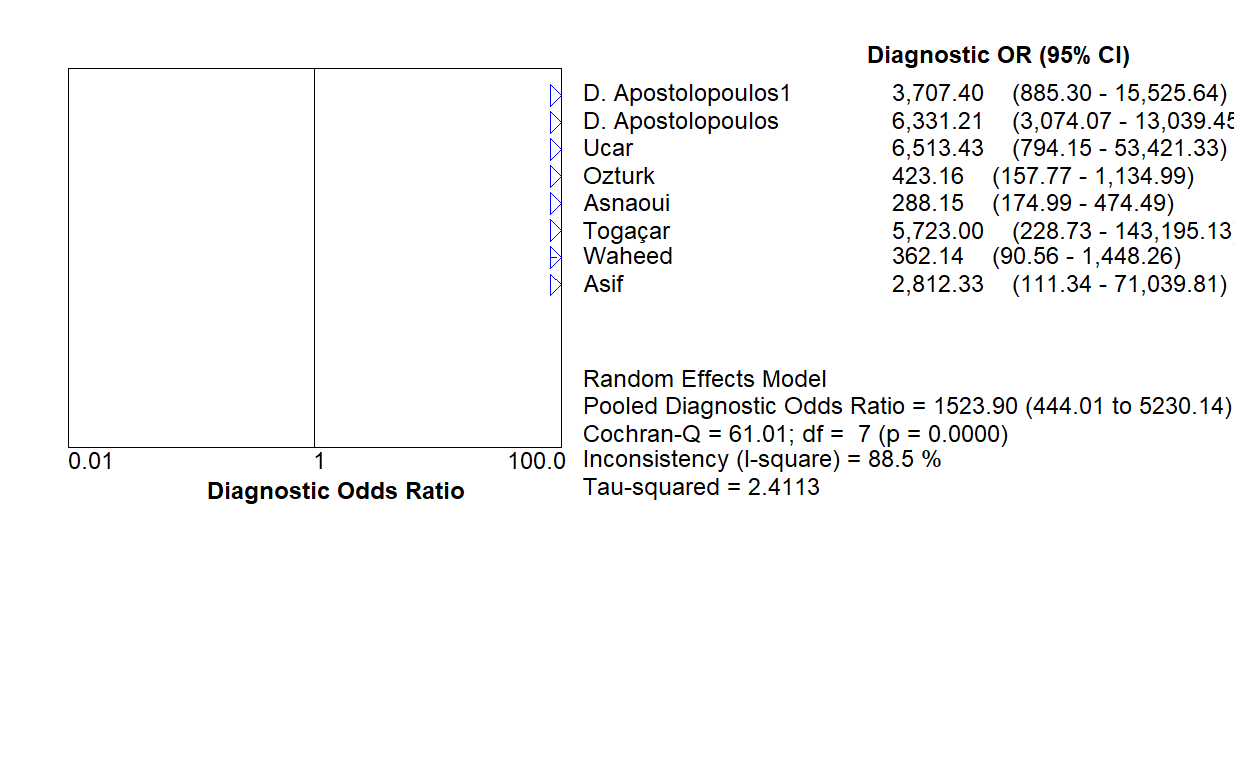


**Figure S5:** Diagnostic odd ratio of DL model for COVID-19 classification using X-ray images.


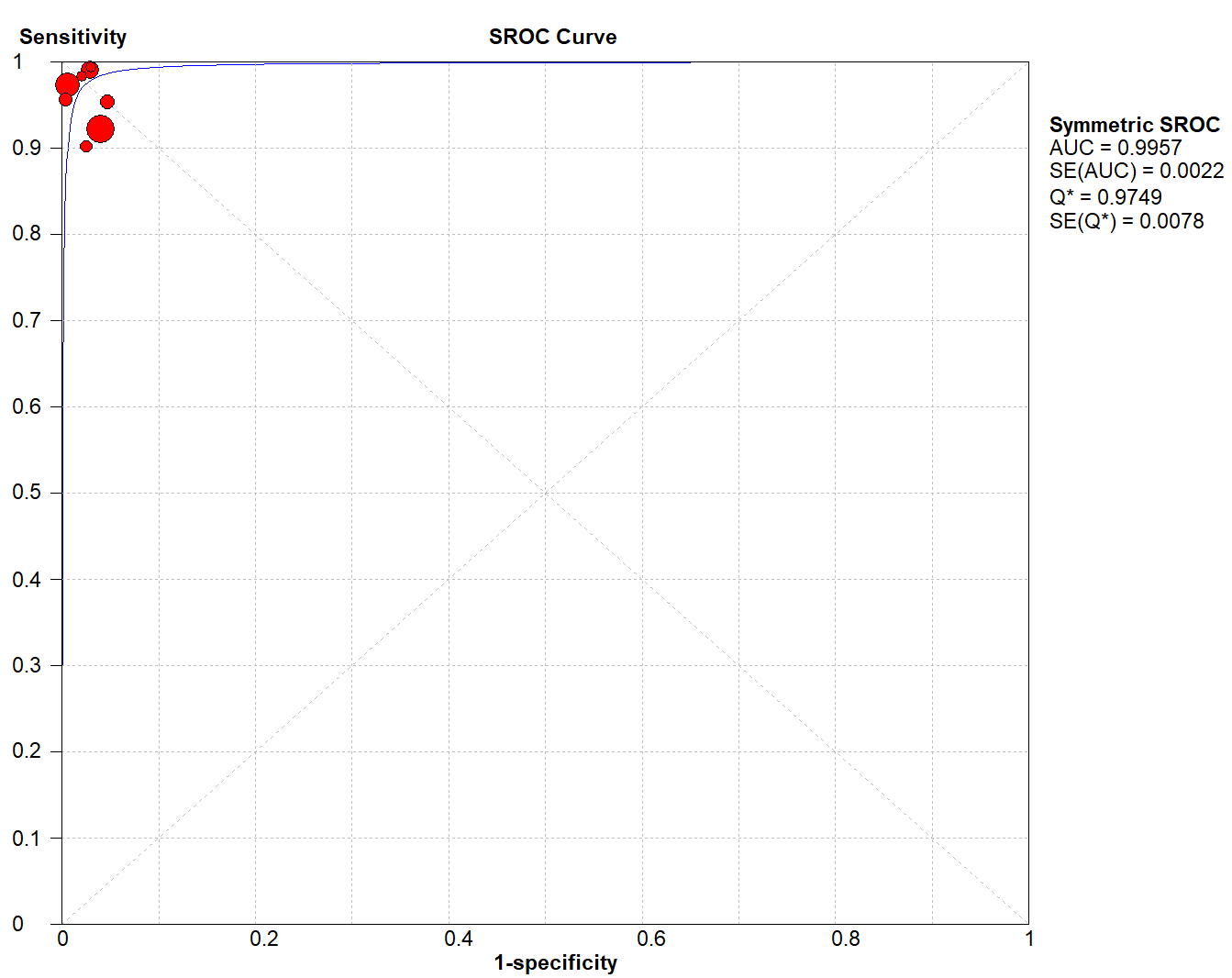


**Figure S6**: AUROC of DL model for COVID-19 classification using X-ray images.


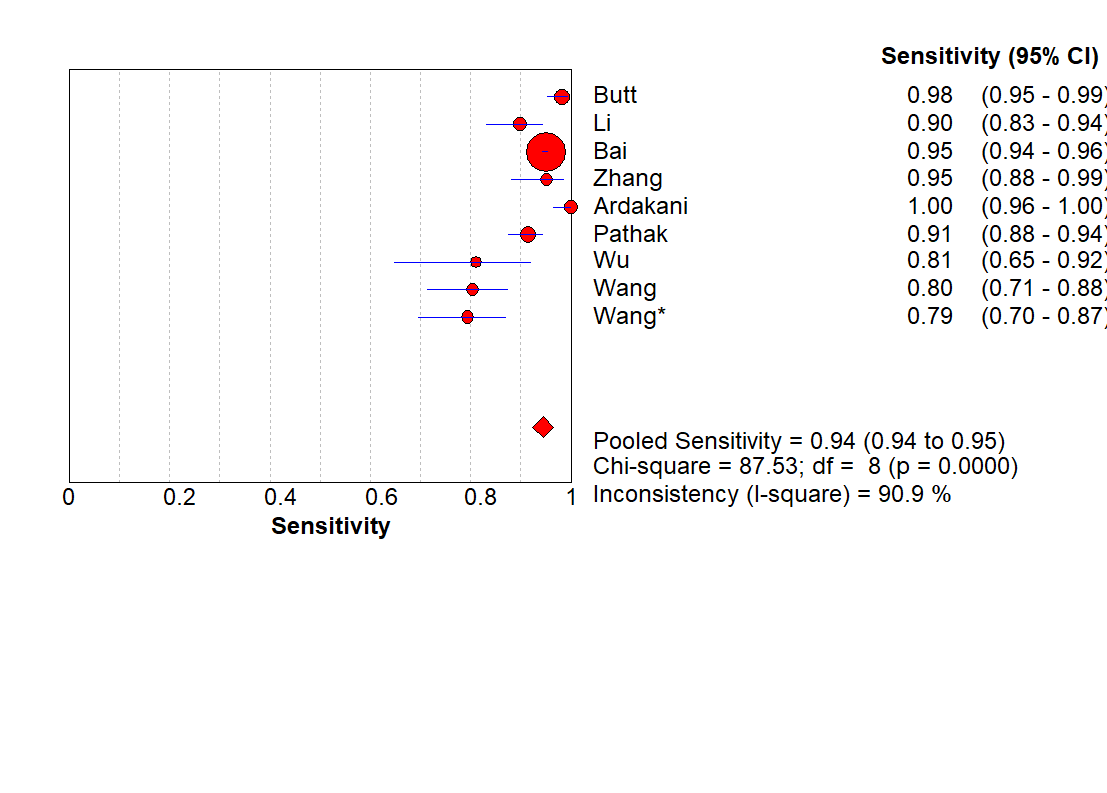


**Figure S7**: Sensitivity of DL model for COVID-19 classification using CT-Scan images.


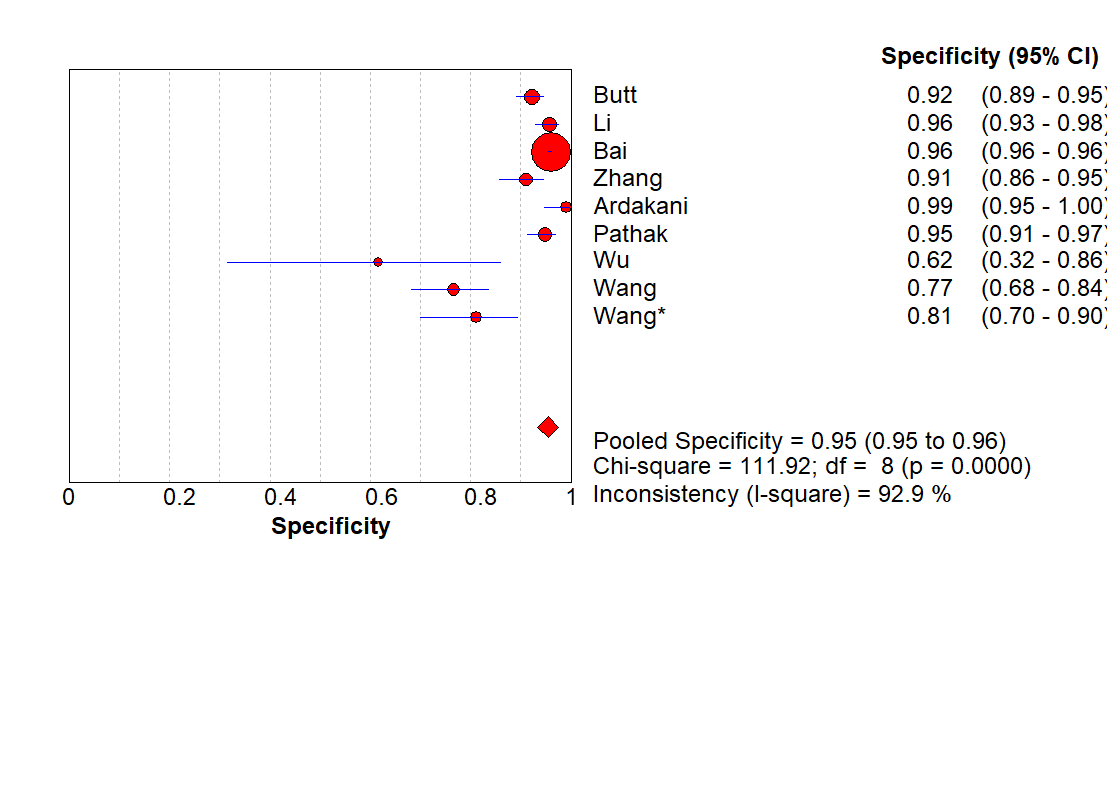


**Figure S8:** Specificity of DL model for COVID-19 classification using CT-Scan images.


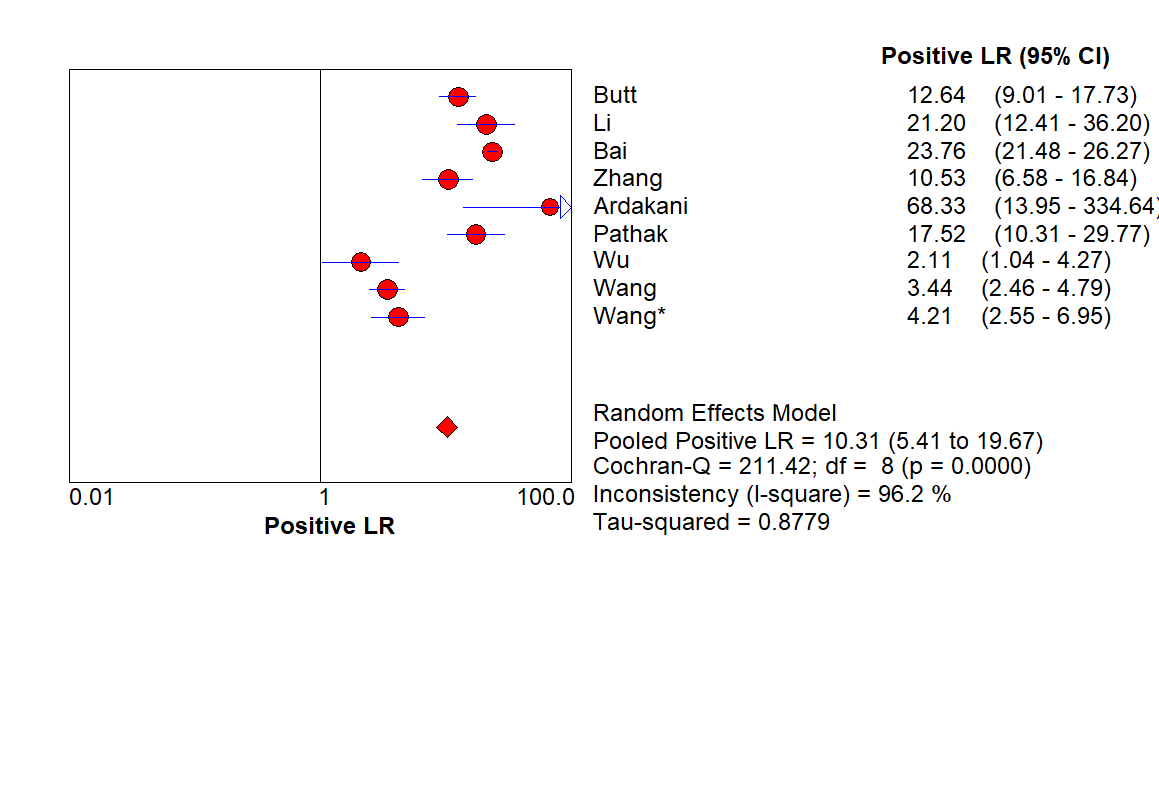


**Figure S9:** Positive LR of DL model for COVID-19 classification using CT-Scan images.


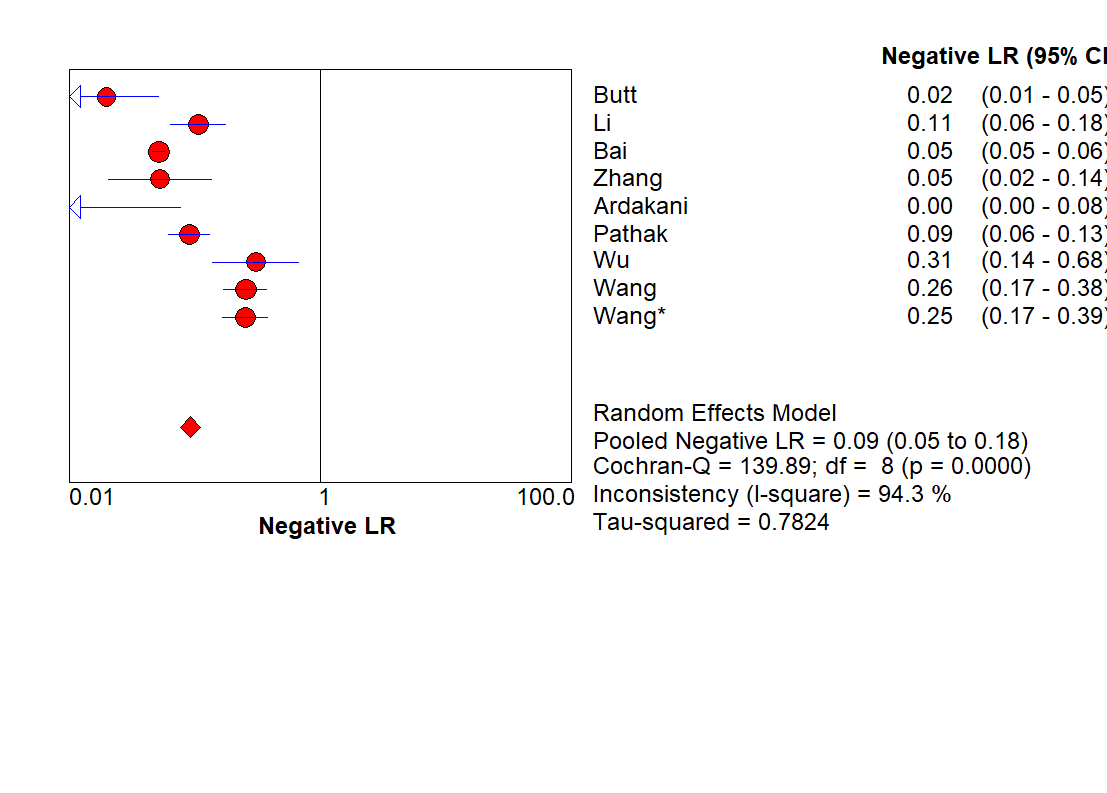


**Figure S10:** Negative LR of DL model for COVID-19 classification using CT-Scan images.


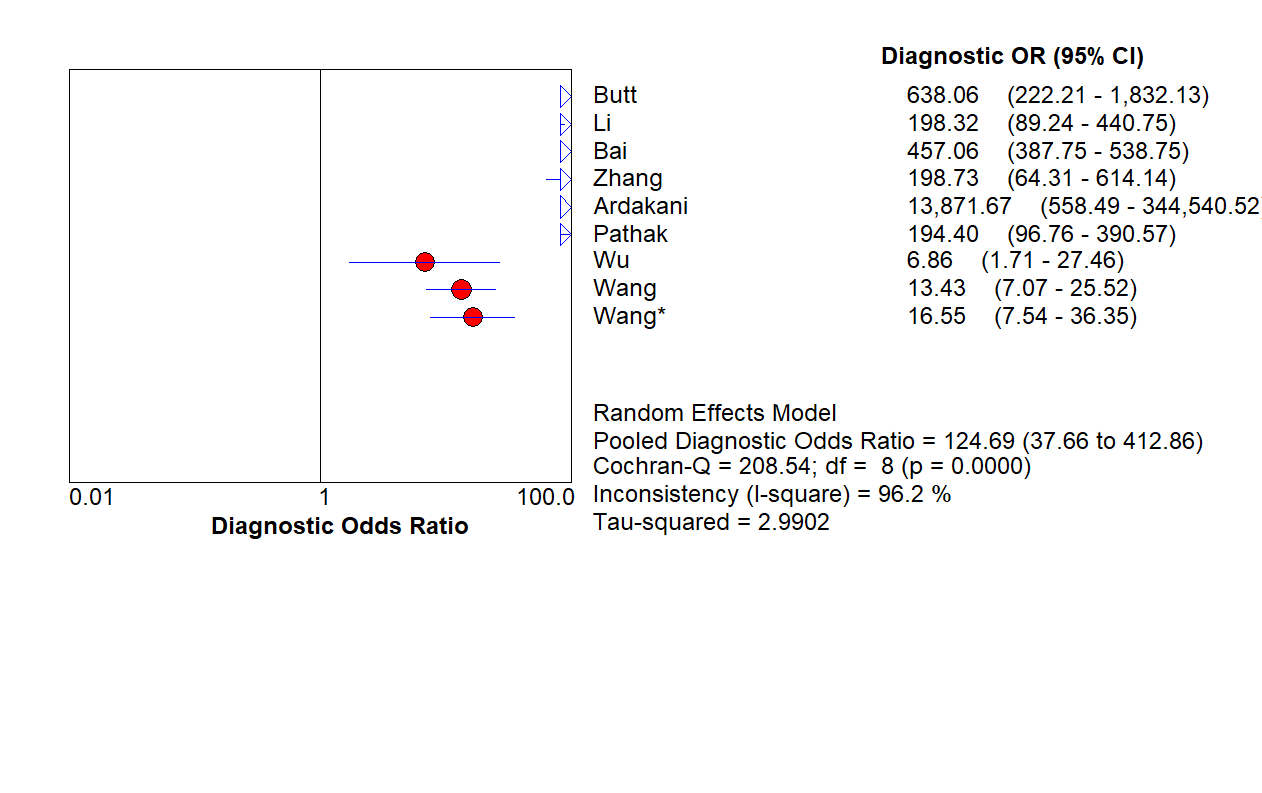


**Figure S11:** Diagnostic odd ratio of DL model for COVID-19 classification using CT-Scan images.


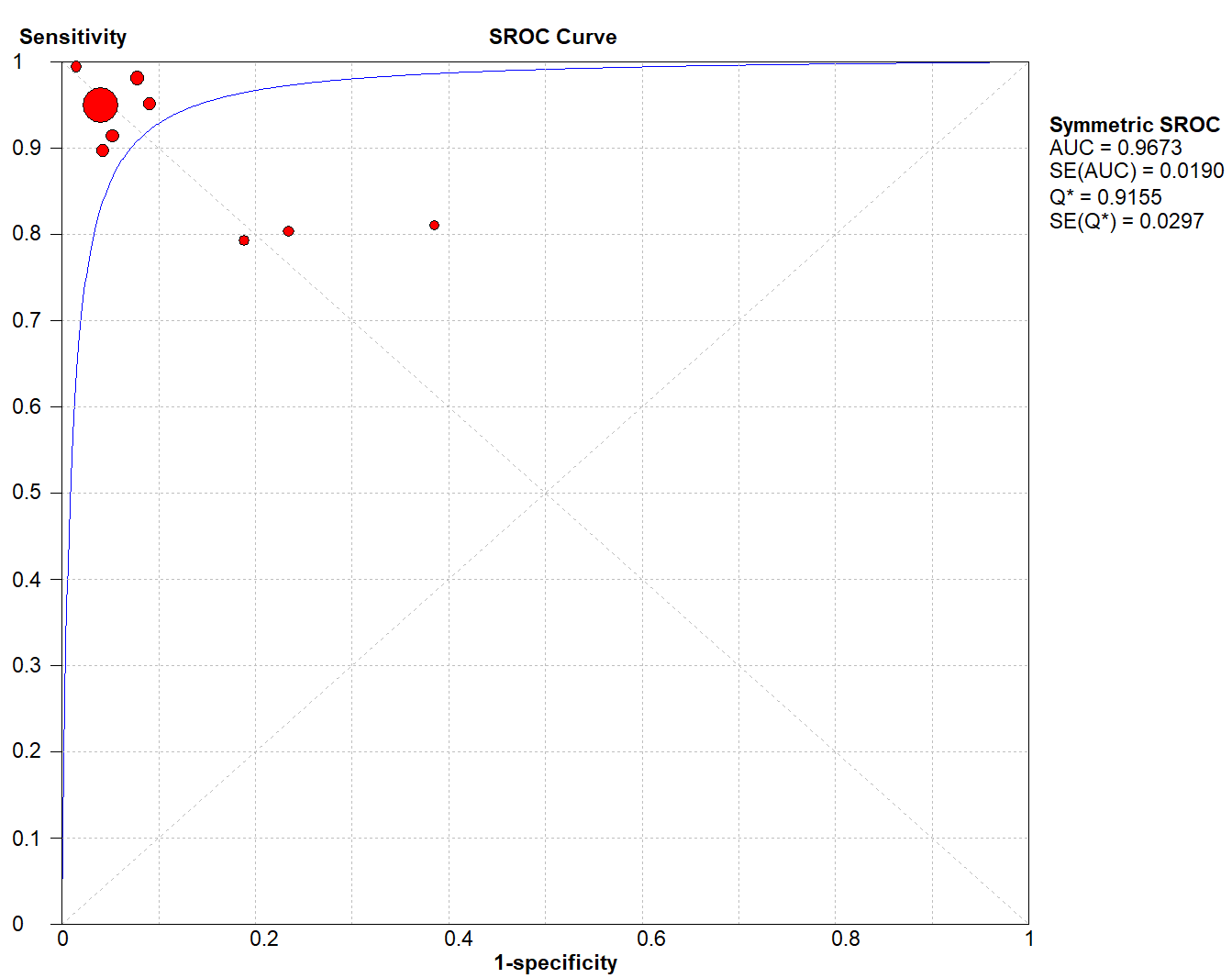


**Figure S12:** AUROC of DL model for COVID-19 classification using CT-Scan images.
